# Genome wide association study of a Circadian Imbalance Index in 312,935 European ancestry UK Biobank participants identifies loci related to diabetes, mood and myocardial infarction

**DOI:** 10.21203/rs.3.rs-8329341/v1

**Published:** 2025-12-12

**Authors:** Magdalena Żebrowska, Matthias Wielscher, Jing Zhang, Ingvild Saksvik-Lehouillier, Lee DiMilia, Angus Burns, Jesse Valliere, Leonardo Vincenzi, Susan Redline, Olivia I Okereke, Richa Saxena, Rebecca Richmond, Martin Rutter, Eva Schernhammer

**Affiliations:** Medical University of Vienna; Medical University of Vienna; Nanjing Medical University; Norwegian University of Science and Technology; Central Queensland University; Brigham and Women’s Hospital, Harvard Medical School; Center for Genomic Medicine, Massachusetts General Hospital, Boston, MA 02114;; Medical University of Vienna; Brigham and Women’s Hospital; Department of Psychiatry, Massachusetts General Hospital and Harvard Medical School, Boston, MA, 02215, USA; Massachusetts General Hospital; University of Bristol; University of Manchester; Department of Epidemiology, Center for Public Health, Medical University of Vienna, Vienna, Austria

**Keywords:** Circadian Imbalance Index, circadian disruption, GWAS, shift work, effect modification

## Abstract

The Circadian Imbalance Index (CII) integrates chronotype, sleep duration, neuroticism, caffeine intake, and vitamin D. In a genome wide association study (GWAS) of CII in 312,935 European ancestry UK Biobank participants, we identified 27 loci mapping to 72 genes, including circadian regulators CALCA, DHCR7, KDM5A, HAL, and CRX. Gene-overlap analyses demonstrated shared architecture with CII components, while EPHB1, SERPING1, C12orf74, PLEKHG7, and EEA1 were uniquely associated with CII. A CII polygenic score (CII-PRS) showed phenome-wide associations with type 2 diabetes (T2D), major depressive disorder, and obesity. Genetic correlations linked CII with insomnia, mood symptoms, body mass index (BMI), T2D, coronary artery disease (CAD), and myocardial infarction (MI). Mendelian randomization suggested causal effects of CII on T2D, mood swings, and MI, and reverse effects of CAD, mood, and MI on CII. This work shows that circadian imbalance is a polygenic trait connecting sleep-related biology to metabolic, cardiovascular and mood health outcomes.

## Introduction

The human circadian system is an internal timekeeping network, with the suprachiasmatic nucleus (SCN) acting as the master pacemaker^1,2^ that synchronizes physiological and behavioral functions to the 24-hour light–dark cycle^3^ via rhythmic expression of clock genes. Together with the homeostatic sleep process, it forms the two-process model of sleep^4,5^, which regulates sleep timing and duration. When these circadian and homeostatic processes are synchronized, sleep-wake cycles remain aligned, whereas if they are desynchronized – circadian misalignment occurs^4,5^.

Epidemiological studies have linked circadian misalignment to a wide array of adverse health outcomes, including impairments in sleep, metabolism, mental health, cognition, cardiovascular functions and immune response^5–8^. Disruptions in circadian rhythms and sleep arise from complex interactions among environmental, behavioral, and genetic factors^5,9–12^, which collectively contribute to individual’s susceptibility. To better understand individual predisposition to circadian misalignment and its association with disease outcomes we recently developed an additive measure - the Circadian Imbalance Index (CII)^13^ - that incorporates five factors: eveningness, short or long sleep duration, low serum vitamin D levels, no or high caffeine consumption, and high neuroticism. Among UK Biobank participants, we identified a strong dose–response relationship between the Circadian Imbalance Index (CII) and the risk of cardiovascular-kidney-metabolic (CKM) disease, especially among European ancestry participants^13^ engaged in shift or night shift work. This suggests a synergistic effect between elevated CII and environmental exposure to circadian disruption. These findings highlight the critical role of circadian traits in human health and diseases risk.

Multiple genetic studies reveal significant overlap between the five components of the CII. Evening chronotype has been found to be genetically associated with increased risk for psychiatric disorders such as depression, anxiety, bipolar disorder, and schizophrenia, mainly mediated by difficulty awakening and circadian misalignment^14^. Clock genes (e.g., PER2, RGS16) are linked with chronotype and are also implicated in obesity and type 2 diabetes through disrupted circadian regulation^15^. Genetic variants related to short sleep duration have been causally linked to chronic widespread pain and poor cardiometabolic outcomes^16^. Neuroticism has been found to be strongly genetically correlated with both insomnia and short sleep duration, and partially with chronotype, suggesting shared biological roots in circadian and stress-related pathways^14^. Multiple studies have also demonstrated that vitamin D modulates circadian regulation through genetic pathways, including the synchronization of core clock genes such as BMAL1, PER2, and NPAS2^17^, transcriptional activation via the vitamin D receptor PDIA3 downstream of Clock^18^, and modulation of osseointegration-related circadian pathways under conditions of vitamin D deficiency^19^, with supplementation shown to restore peripheral clock gene expression in bone marrow stromal cells^20^. Further, caffeine intake may delay the circadian clock in humans^21,22^. It also alters expression of core clock genes (Clock, Per2, Cry1, Cry2)^23,24^, behavioral rhythms and peripheral clock gene synchronization^25^, demonstrating its molecular effects on circadian regulation.

However, to the best of our knowledge, no study has analyzed the combined genetic effects of all five CII components within a single integrative framework. Here we report a large-scale genome-wide association study (GWAS) of CII using a European discovery cohort of N = 312,935 UK Biobank participants with replication in an independent population of N = 11,344 European ancestry women from the Nurses’ Health Study II (NHS2)^26^. We then assessed effect modification by sex and by shift-work status, conducted two phenome-wide association studies (PheWAS) of the CII polygenic risk score (PRS) among European ancestry individuals in a clinical biobank (the Mass General Brigham Biobank^27^; MGBB, N = 50,908) and a population-based cohort (All Of US^28^; AoU, N = 98,182). PheWAS-significant disease outcomes were then subsequently checked for its genetic correlation with the CII in the UKB, and those which showed significant genetic correlations were used in further Mendelian randomization analysis to test for putative causal relationships. This work identifies genetic drivers of circadian imbalance and clarifies sex- and shift-work–specific effects, enabling more precise risk stratification and informing targeted prevention strategies for cardiometabolic and psychiatric disorders linked to disrupted circadian biology.

## Results

Table 1 presents baseline characteristics of the study participants, stratified by levels of the CII and summaries for each of the CII’s components. A total of 312,935 European ancestry individuals with complete genotype, CII, and covariates data were included. Participants had a mean age of 56.6 years (SD = 8.1), a mean body mass index (BMI) of 27.4 kg/m^2^ (SD = 4.7) and were 47.4% male. Those with higher CII were less likely to be men (40.5% within CII = 5 vs 53.5% in CII = 0), have higher BMI (29.2 (SD = 5.9) in CII = 5 vs 26.4 (SD = 3.9) in CII = 0) and more likely to be involved in shift work (26.3% in CII = 5 vs 13% in CII = 0). Individuals with higher CII were also more likely to have an average total household income of less than £18,000 (33.3% in CII = 5 vs 14.7 in CII = 0) and less likely had college or university degree (23.9% in CII = 5 vs 35.7% in CII = 0).

### Genome-wide association studies in the UKB

**Fig. 1:**
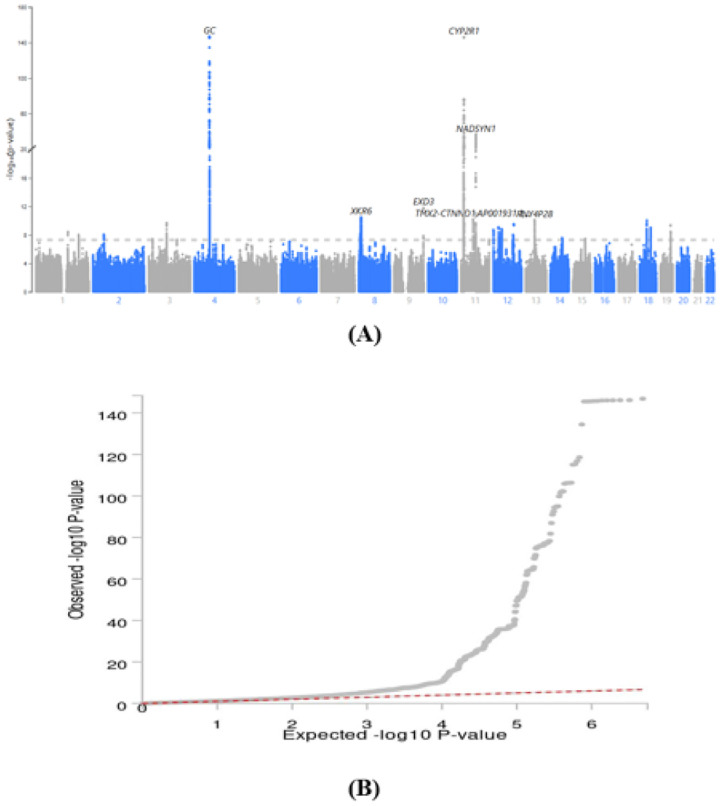
Manhattan plot (A) and quantile-quantile plot of expected versus observed log10P values (B) from of the genome-wide association study of the CII.

The Manhattan plot (Fig. 1(A)) and quantile-quantile (QQ) plot (Fig. 1(B)), generated using LocusZoom^29^, illustrate GWAS results of the CII. We observed moderate inflation of the test statistics (λ_GC_ = 1.27, mean χ^2^ = 1.34). LD-score regression yielded SNP based heritability of h_SNP_^2^=0.066 (SE 0.0033; Supplementary Table 1) and an intercept of 1.01 (SE 0.0076), suggesting that inflation was predominantly due to polygenicity rather than confounding^30^ (attenuation ratio 0.03).

### Functional consequences of genes

Functional annotation of all SNPs in linkage disequilibrium (LD; r^2^ ≥ 0.6) identified 27 genomic risk loci (Table 2). The most significant genomic loci was rs34265662 on chromosome 4 (p = 1.49E-147, β = 7.93E-02, SE = 0.003) near GC gene followed by rs117913124 on chromosome 11 (p = 1.01E-146; β = 0.22; SE = 0.009) near CYP2R1. Other significant genomic loci were mapped to e.g., NADSYN1 (p = 5.05E-37), EXD3 (p = 1.57E-12) as well as e.g., CELF4 (p = 9.28E-11) or SOX5 (p = 9.12E-10). A total of 72 genes with recognized Ensembl ID were mapped (Supplementary Table 2), with 5 circadian rhythm related genes (CRGs) among them (CALCA, DHCR7, KDM5A, HAL, and CRX; Table 3). The list of CRGs^31^ (Supplementary Table 3) was obtained from MSigDB database^32–34^.

**Fig. 2:**
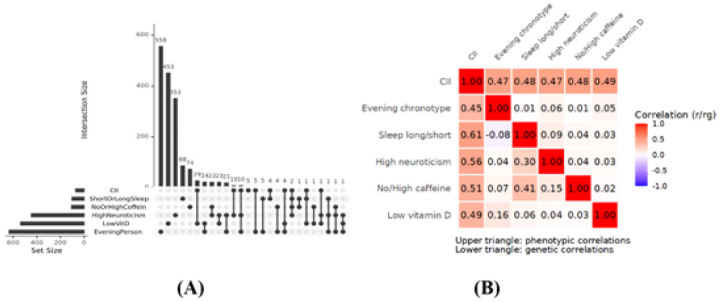
CII and its 5 components: **(A)** Frequency of genes overlap between the CII and its components **(B)** phenotypic (r) and genetic (r_g_) correlations between CII and its 5 components. Genetic correlations were computed using bivariate LD score regression.

When analyzing genetic overlap between the CII and its components we observed 29 genes that determined both CII and low vitamin D levels, 10 genes that determined both CII and high neuroticism, 5 common genes for CII and eveningness, and 4 common genes between CII and short or long sleep duration (Fig. 2(A)). There were no common genes that would determine both CII and no or high coffee consumption. In addition, we identified five genes—EPHB1, SERPING1, C12orf74, PLEKHG7, and EEA1—that were significantly associated with the CII but not with any of its individual components (Table 4).

Tissue expression analysis performed in MAGMA indicated expression in brain cerebellum, frontal cortex and nucleus accumbens (Supplementary Fig. 1) and gene set analysis indicated significant curated gene sets M39352 (vitamin D metabolism; adjusted p-value 0.0326) and M1493 (Nikolsky breast cancer 11q12_q14 amplicon; adjusted p-value 0.0492; Supplementary Table 4). Among GWAS catalog recognized gene sets, some were associated with CII components (Vitamin D levels and insufficiency, neuroticism, sleep duration and chronotype; Supplementary Table 4). In addition, CII was associated with GWAS catalog gene sets linked to psychiatric and neurodevelopmental disorders, cardiometabolic traits, immune-related conditions, behavioral tendencies, and lifestyle factors (Supplementary Table 4).

### Phenotypic and genetic correlations between the CII and its components

To further evaluate the validity of the CII, we examined its phenotypic and genetic correlations with its individual components - and among the individual components themselves - within our study sample. Phenotypically, CII showed a consistent moderate to strong positive correlation with each of the component traits (Fig. 2(B)). Genetic correlations were uniformly stronger than the phenotypic correlations (Fig. 2(B); Table 9), indicating that the shared variance between CII and its components is largely due to genetic factors. The strongest genetic associations were observed for high neuroticism (r_g_ = 0.56, p = 4.79×10^−143^) and short or long sleep duration (r_g_ = 0.61, p = 1.0×10^−117^), while caffeine intake (r_g_ = 0.51, p = 4.33×10^−53^) and low vitamin D levels (r_g_ = 0.49, p = 5.92×10^−82^) also exceeded their phenotypic counterparts. Evening chronotype showed a slightly lower but still strong genetic correlation (r_g_ = 0.45, p = 4.73×10^−88^). This suggests that CII accounts for a substantial portion of the shared genetic susceptibility across component traits, beyond what can be observed at the phenotypic level.

### Genetic-variant—shift work GWAS results

To test whether associations of genetic variants with CII differ by shift-work status we conducted a genetic-variant-by-shiftwork GWAS. Using the default parameters of FUMA we identified 91 GW-significant (p < 5×10^− 8^) interaction lead variants (Table 15). After conducting separate shift-work stratified GWASs (Supplementary Fig. 2), we checked effects of interaction lead variants among shift workers (N = 28,976) and no-shift workers (N = 154,262) (Supplementary Table 5). Since the shift workers (SW) subgroup contained substantially fewer individuals than the no shift workers (noSW), SW-specific effect estimates had larger standard errors, reducing statistical power to detect the interaction. Across the 91 lead variants, interaction p-values were mostly similar to those in the noSW stratum (Supplementary Fig. 3), and the estimated differences in effects were small with wide confidence intervals in SW (Supplementary Table 5, Supplementary Fig. 4), suggesting that the lack of interaction significance largely reflects limited precision in the smaller SW stratum rather than strong evidence against effect modification. Variants with both opposite effect size directions and heterogeneity p-values that remain significant after multiple-testing correction (α = 0.05/91 = 5.5×10^−4^), were considered as indicating SW-specific effect modification. Of the 91 lead variants, two showed significant heterogeneity— rs79799991 (5:141914736:A:T; P_delta_=1.9×10^−4^; P_int_=9.6×10^−6^) close to FGF1 gene and rs7161954 (15:78118955:G:T; P_delta_=2.4×10^−4^; P_int_=1.9×10^−7^) close to LINGO1 gene —both indicating smaller effects in SW than noSW (Supplementary Table 5).

### Sex differences in genetic associations

We conducted genetic-variant-by-sex and sex-specific GWAS analyses for the CII in our study sample to investigate potential modification of genetic variants effects by sex (Supplementary Fig. 5). Compared to the overall sample analysis, of the five CII-specific genes (Table 4), three (EPHB1, PLEKHG7 and EEA1) were also identified in women-specific FUMA results (Table 4) while none of them were found in men-specific analyses. When looking at corresponding independent significant SNPs for the five CII-specific genes, they are placed on three genomic loci (chr 3 (GL 6), chr 11 (GL 12) and chr 12 (GL 20); Table 4). Two of these three loci were also identified among women (chr 3:134729537:C:G (EPHB1) and chr12:93222395 :C:CAT (EEA1)) with one (chr3:134729537:C:G; rs4082244) showing the same direction of effects in both overall (β=−0.0160, se = 0.0029) and women-specific analyses (β=−0.0303, se = 0.0058; Table 4).

Using the default FUMA parameters we obtained 65 significant lead variants for SNP-sex interaction (Supplementary Table 6). Based on conducted sex-stratified GWASs we compared effect sizes for the identified genetic-variant-by-sex interaction lead SNPs between men (N = 148,473) and women (N = 164,462; Supplementary Table 7, Supplementary Fig. 6) and their p-values (Supplementary Fig. 7). There were nine variants (Supplementary Table 8) with opposite effects directions in men and women and heterogeneity p-values that remain significant after multiple-testing correction (α = 0.05/65 = 7.7×10^−4^), which we considered as indicating significant effect modification by sex. These nine variants are located near genes linked to processes such as lipid and glucose metabolism (PID1), blood clotting and vessel repair (SERPINE1), inflammation (ALOX5AP), and cell growth signaling (NXN) (Supplementary Table 9). Tissue expression analysis (MAGMA) for SNP×sex interaction analysis did not identify any differentially expressed genes, whereas the gene-set analysis revealed trait enrichments that were not detected in the overall GWAS, such as blood pressure interaction tests with alcohol and smoking, body-shape indices, neurobehavioral and reproductive traits (loneliness, risk tolerance, cognitive function, age at first sex, number of partners), brain structure (pallidum volume, brain morphology, chronic back pain), ocular and vascular phenotypes (pathological myopia, retinal caliber), autoimmune disease (systemic sclerosis, systemic lupus), bone measures and a locus at chr6p21 (Supplementary Table 10). In contrast, traits overlapping between SNP×sex and the overall GWAS clustered around vitamin D biology and neuroticism-related phenotypes (neuroticism, mood instability, depression, SSRI-remission), with additional overlaps for refractive error, heart-rate response to exercise, risk-taking, chromosomal aberration burden, diastolic blood pressure×smoking status and loci at chr8p23, chr1q21, and chr11p15. These patterns indicate strong sex-dependent genetic enrichment for blood pressure interaction with lifestyle and body-shape traits, whereas vitamin D and neuroticism signals are largely shared across sexes (Supplementary Table 10).

### PheWAS of the CII-PRS in a clinical and population-based biobanks

**Fig. 3:**
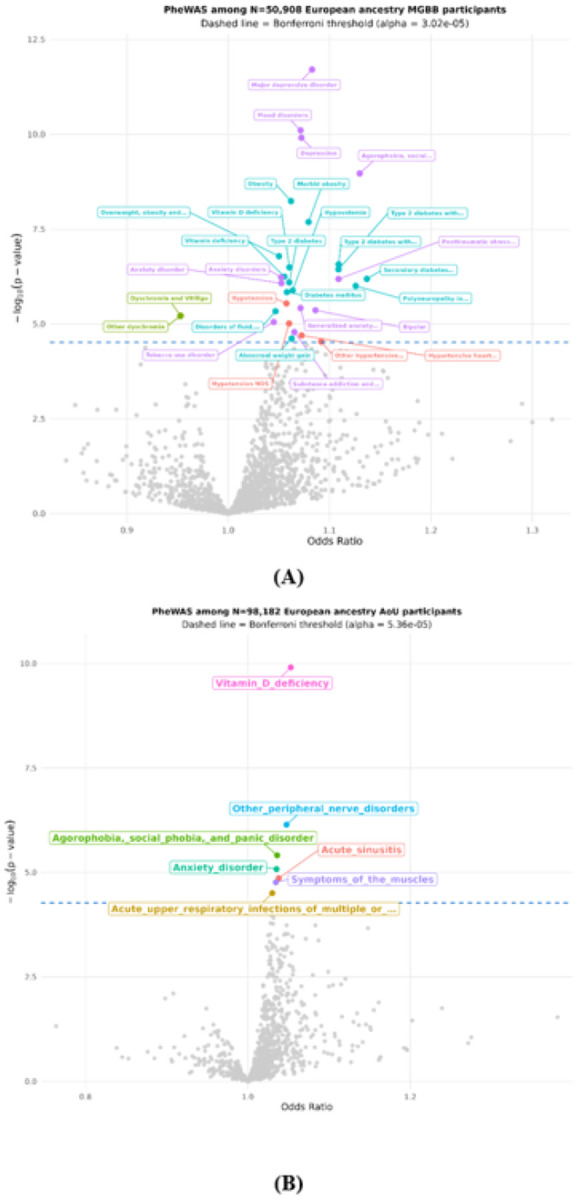
Results of the PheWAS analysis of the PRS-CS polygenic risk score for the CII (CII-PRS) among European ancestry **(A)** N=50,908 MGB Biobank participants **(B)** N=98,182 European ancestry AoU participants.

We further searched for CII-associated phenotypes by conducting two PheWAS of a CII polygenic risk score (PRS) generated with PRS-CS^35^. The PRS weights were trained in the UK Biobank and then applied to compute CII-PRS among N = 50,908 European ancestry individuals in the MGBB and among N = 98,182 European ancestry participants in the AoU. The PheWAS in the clinical MGBB identified thirty-one disease phenotypes significantly (p_MGBB_<3.02E-5) associated with the CII-PRS (Supplementary Table 11, Fig. 3(A)) with the highest odds-ratios (OR) observed for endocrine or metabolic disorders (secondary diabetes mellitus, OR = 1.14, 95%CI=(1.08, 1.20); polyneuropathy in diabetes, OR = 1.13, 95%CI=(1.07, 1.18); type 2 diabetes with renal manifestations, OR = 1.11, 95%CI=(1.07, 1.15); type 2 diabetes with neurological manifestations, OR = 1.11, 95%CI=(1.07, 1.15)), mental disorders (agorophobia, social phobia, and panic disorder, OR = 1.13, 95%CI=(1.09, 1.18); posttraumatic stress disorder, OR = 1.11, 95%CI=(1.07, 1.16); bipolar, OR = 1.09, 95%CI=(1.05, 1.13); major depressive disorder (MDD), OR = 1.08, 95%CI=(1.06, 1.11)), circulatory system (hypertensive complications, OR = 1.09, 95%CI=(1.05, 1.14); hypertensive heart or renal disease, OR = 1.07, 95%CI=(1.04, 1.11)) or dermatologic (dyschromia and vitiligo, OR = 0.95, 95%CI= (0.93, 0.97)) disorders. Subsequent sex specific PheWAS analyses in the MGBB indicated associations with obesity, mood disorders, depression, anxiety and skin cancer for women (Supplementary Table 12, Supplementary Fig. 8(A)) and angina pectoris and ischemic heart disease for men (Supplementary Table 13, Supplementary Fig. 8(B)).

In the AoU cohort, there were 7 diseases phenotypes significantly associated (p_AoU_<5.36E-05) with the CII-PRS (Supplementary Table 14, Fig. 3(B)) with the highest ORs observed for Vitamin D deficiency (OR = 1.05, 95%CI=(1.04, 1.07)) followed by peripheral nerve disorders (OR = 1.05, 95%CI=(1.03, 1.07)), acute sinusitis (OR = 1.04, 95%CI=(1.02, 1.06)), agoraphobia, social phobia and panic disorder (OR = 1.04, 95%CI=(1.02, 1.05)), anxiety disorder (OR = 1.04, 95%CI=(1.02, 1.05)), symptoms of the muscles (OR = 1.03, 95%CI=(1.02, 1.05)), and acute upper respiratory infections of multiple or unspecified sites (OR = 1.03, 95%CI=(1.02, 1.04)).

### CII-associated phenotypes and genetic correlation analysis

**Fig. 4:**
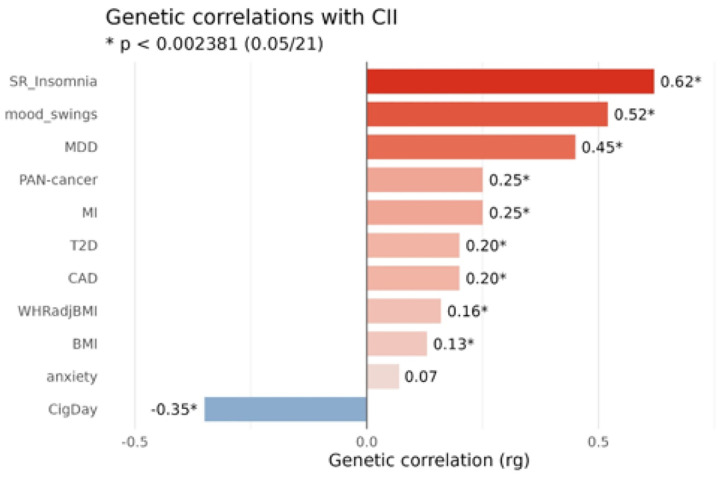
Genetic correlations between the CII and (disease) phenotypes of interest.

We created an integrated list of (disease) phenotypes of interest, including GWAS-catalog traits and gene sets identified by FUMA (Supplementary Table 4), as well as disease phenotypes significantly associated with CII-PRS in the MGBB or AoU PheWAS analyses (Supplementary Table 11, Supplementary Table 14). We excluded CII components and phenotypes with low heritability or case numbers. The final list contained sixteen traits and disease phenotypes (Supplementary Table 15) for which we used publicly available UKB-GWAS-based summary statistics to estimate their genetic correlation with the CII. Significance level for genetic correlation was corrected for multiple testing (16 diseases plus 5 components: p < 0.05/21 = 0.0023). Significant positive genetic correlations were observed between CII and self-reported insomnia (r_g_=0.62, p = 1.22E-156), MDD (r_g_=0.45, p = 5.99E-16), mood swings (r_g_=0.52, p = 2.00E-92), myocardial infarction (MI; r_g_=0.25, p = 2.74E-20), coronary artery disease (CAD; r_g_=0.2, p = 6.34E-10), T2D (r_g_=0.2, p = 5.87E-23), BMI (r_g_=0.13, p = 2.85E-06), BMI-adjusted waist-to-hip-ratio (r_g_=0.16, p = 3.39E-06) and (borderline) for cancer (r_g_=0.25, p = 0.002). Furthermore, CII was significantly negatively correlated with genetically instrumented number of cigarettes smoked per day (r_g_=−0.35, p = 4.70E-30; Fig. 4; Supplementary Tables 16–17).

### Two sample Mendelian randomization

We considered the ten traits which showed significant genetic correlation with CII (insomnia, mood swings, MDD, cancer, MI, T2D, CAD, WHRadjBMI, BMI, no. cigarettes) and examined potential causal effects for their associations with the CII. Summary statistics for these phenotypes were obtained from FinnGen^36^ (insomnia, mood, cancer, MI, T2D, CAD, BMI), The Psychiatric Genomics Consortium (MDD^37^), GIANT (WHRadjBMI^38^) and Tobacco and Genetics Consortium (cigarettes per day^39^; Supplementary Table 18). We performed bi-directional two sample Mendelian randomization using GSMR^40–42^. In the forward direction (CII being an exposure and the ten phenotypes being outcomes), GSMR provided evidence that higher CII was associated with increased odds of T2D (OR = 1.170; 95%CI=(1.053, 1.299)), MI (OR = 1.208; 95%CI=(1.029, 1.418)) and mood disorder (OR = 1.129; 95%CI= (1.010, 1.261)) (Supplementary Table 19). Instrument selection used genome-wide significant variants (P < 5×10^−8^) clumped for independence (LD r^2^ < 0.05), and potential horizontal pleiotropy was addressed using the HEIDI-outlier procedure (threshold P < 0.01), removing variants with evidence of pleiotropic effects. In the CII to T2D analysis, clumping produced 25 independent index SNPs and 2 were removed by HEIDI-outlier filtering, leaving 23 instruments (multi-SNP HEIDI-outlier P = 0.1058). In the CII-to-mood analysis, clumping yielded 25 index variants and HEIDI filtering removed 2 outliers, leaving 23 instruments with a multi-SNP HEIDI-outlier P-value of 0.046. In the CII-to-MI analysis, 25 index variants were retained after HEIDI filtering (no outliers removed), with multi-SNP HEIDI-outlier P = 0.717 (Supplementary Table 20).

In reverse direction, genetically predicted MI (β = 0.01; SE = 0.005, p = 0.034), CAD (β = 0.02; SE = 0.005; p = 0.002) and mood (β = 0.13; SE = 0.019; p = 8.32E-12) were causally associated with CII. In the mood-to-CII analysis, 21 index variants were identified, 2 were removed as HEIDI outliers, and 19 instruments remained, with multi-SNP HEIDI-outlier P = 0.145. In MI-to-CII, 70 index variants were identified, 4 were removed as HEIDI outliers, and 66 instruments remained, with multi-SNP HEIDI-outlier P = 0.167. For CAD-to-CII, clumping identified 132 index variants, 7 were removed as HEIDI outliers, and 125 instruments remained for GSMR (multi-SNP HEIDI-outlier P = 0.158; Supplementary Table 20).

Overall, these results suggest evidence of effects in both directions for mood and MI, while HEIDI-based filtering removed a small subset of variants flagged as pleiotropic and multi-SNP HEIDI-outlier P-values did not generally indicate pervasive pleiotropy across retained instruments.

### Replication in the Nurses’ Health Study II

For replication analysis we used 11,351 women data from the NHS2 cohort. Compared to UKB women, nurses in the NHS2 were on average younger (56.2 (8.0) in the UKB vs 55.39(4.40) in NHS2) and had similar average BMI (27.0 (5.1) in the UKB vs 27.34(6.22) in NHS2). In both cohorts, women with higher CII values were more likely to have higher BMI, lower socio-economic status and were more likely to perform shift work (Supplement 4, Tables N2–N3). In our recent study^13^ in the UKB, CII was associated with an increased cardio-kidney-metabolic (CKM) risk. Particularly, among individuals of European ancestry, high CII posed the greatest risk if combined with night shift work. We conducted a series of phenotypic association analyses to determine how well the CII constructed in NHS2 replicates associations observed in the UKB. Following our analyses in Zhang et al^13^., we also investigated whether the cumulative duration of shift work modified the association between CII and the CKM.

The analysis of association between the CII and the CKM in the NHS2 indicated increasing CKM risk with higher CII levels and significant increasing trend (p_trend_<.0001; Supplement 4, Table N4). Furthermore, similarly as in the UKB, the risk of the CKM increased with the higher CII level and duration of shift work, with significant trends in each of the three CII levels (Low (0–1), middle (2–3), high (4–5); Supplement 4, Tables N5–N7). Interestingly, in the NHS2 this trend was observed already when considering cumulative duration of shift work categorized into never, less than 5 years, 5 or more years of shift work, whereas in the UKB it was only observed under categorization :never, less than 20 years of shift work, 20 or more years of shift work. This can be attributed to considerably larger sample of nurses performing shift work and good quality assessment of this phenotype in the NHS2. In summary, these phenotypic replication analyses confirm that the CII derived in the NHS2 shows characteristics and associations patterns similar to the ones observed in the UKB.

Since the NHS2 is a women-only cohort, GWAS replication was performed for lead SNPs obtained from UKB-women only GWAS (N = 164,462). Of the 104 genome-wide significant (GWS) lead variants among UKB women, 84 were present in the NHS2 after QC (Supplementary Table 21), and ten of them demonstrated nominal significance in the NHS2 (p < 0.044; Supplementary Table 22). Four of them (rs11917139 (ZBTB20), rs330905 (PPP1R3B), rs35358081 (OR1J1), rs10848644 (SLC6A13)) also showed consistent effect directions in both discovery and replication cohorts.

We further analyzed predictive properties of the CII polygenic risk score (PRS) generated with PRS-CS^35^. The PRS weights were trained in the UK Biobank and were applied to compute CII-PRS in the NHS2. We evaluated the association between the normalized (z-scored) polygenic risk score for CII (zCII-PRS) and the observed CII among 11,344 women in the NHS2. We interpreted CII as a continuous measure and z-scored it to assess normality and analyzed its association with zCII-PRS using linear models adjusted for age and 10 genetic principal components. Addition of zCII-PRS to the model significantly improved model fit compared with a base model including age and ancestry principal components only (likelihood ratio χ^2^ = 16.3, p = 5.4×10^−5^), although the increment in explained variance was modest (< 0.2%).

## Discussion

In this study, we investigated the genetic architecture of a newly developed Circadian Imbalance Index, designed to enhance understanding of circadian phenotypes and their common genetic basis.

In the GWAS analysis of the CII we identified 27 genomic risk loci and 72 associated genes, including five previously known to be involved in circadian regulation (CALCA, DHCR7, KDM5A, HAL, and CRX). The strongest association was observed near genes associated with Vitamin D binding protein and 25-hydroxyvitamin D (GC, CYP2R1), metabolic and endocrine pathways (PDE3B, PSMA1, RRAS2)^43–45^, neurovascular signaling and pain (CALCA, CALCB)^46–48^, cellular maintenance systems (COPB1, PSMA1)^49^ and cholesterol and Vitamin D metabolism (NADSYN1, DHCR7)^50–53^.

Further, we found that five other genes—EPHB1, SERPING1, C12orf74, PLEKHG7, and EEA1—were significantly associated with the CII but not with any of its individual components. Of them, the PLEKHG7 shows indirect evidence of association with circadian disruption through a related gene in the same family - PLEKHG6 - which has been found to undergo epigenetic changes in response to circadian disruption, particularly in the context of maternal night shift work^54^. The protein product of the SERPING1 gene is structurally related to PAI-1 (Plasminogen Activator Inhibitor-1, encoded by SERPINE1), a serpin with a known role in circadian regulation. Although SERPING1 itself has not been directly linked to circadian gene regulation, its structural similarity to PAI-1 suggests that SERPING1-related pathways may plausibly modulate circadian rhythms^55^. For EPHB1, C12orf74 and EEA1 genes, to the best of our knowledge there is currently no literature findings connecting these genes to circadian rhythm regulation. EPHB1 is a receptor tyrosine kinase involved in neural development, including axon guidance at the optic chiasm for proper retinal ganglion cell projection^56^, as well as immune cell maturation and cell migration^57,58^; its reduced expression is linked to increased tumor invasiveness and poor prognosis in colorectal, gastric, ovarian, and brain cancers^59–61^, with additional associations to chronic muscle pain^62^. C12orf74 has been identified as a novel oncogene that is upregulated in lung adenocarcinoma and cervical cancer, where it promotes tumor proliferation and metastasis through activation of the EGFR/AKT/mTORC1 signaling pathway and is associated with poor prognosis, particularly in HPV-positive cervical tumors due to promoter hypomethylation^63^; in addition, C12orf74 has been linked to reduced coronary artery disease risk^64^. Variants and altered expression of EEA1 have been linked to several diseases, including increased susceptibility to fungal infection in allergic bronchopulmonary aspergillosis^65^, autoimmune conditions like lupus where it acts as an autoantigen^66,67^, and neurological disorders^68^.

We further identified 65 variants with sex-specific associations, nine of which showed opposite effects in men and women, implicating pathways related to metabolism, vascular biology, inflammation, and cell signaling. These results suggest that biological sex not only influences the prevalence of circadian disruption but also modifies the impact of underlying genetic variants. In addition, we observed 91 lead variants with evidence of interaction with shift-work status, a key environmental determinant of circadian disruption. Although statistical power was limited in the smaller shift work subgroup, two variants— rs79799991(FGF1) and rs7161954 (LINGO1)—showed heterogeneity, with smaller effects among shift workers compared to non–shift workers. FGF1 is key in metabolic regulation and disease. In the pancreas, disruption of FGF1/FGFR1 signaling leads to impaired insulin processing, contributing to diabetes-like phenotypes^69^. FGF1 is also co-expressed with FGFR1 in melanoma and breast cancer^70,71^ and it supports adipose tissue remodeling^72^ highlighting its role in metabolic homeostasis and potential as a therapeutic target. LINGO1 is critical for CNS development, with variants linked to higher risk and altered phenotypes in essential tremor and Parkinson’s disease. Together, these findings highlight that both sex and occupational exposures can shape how genetic variation contributes to circadian imbalance. Incorporating such effect modifiers into future genetic studies will be essential for refining risk prediction and understanding the mechanisms linking circadian disruption to disease.

Across two independent resources—a hospital-based biobank (MGBB) and a population cohort (AoU)—the CII-PRS showed phenome-wide associations with neuropsychiatric, metabolic, and cardiometabolic domains. In MGBB, the strongest associations were to endocrine/metabolic diseases (T2D, hypertensive complications) and mental disorders (MDD, mood disorders, depression), In AoU, anxiety-spectrum diagnoses, peripheral nerve disorders, musculoskeletal symptoms, acute sinusitis, and upper respiratory infections were observed. Sex-stratified PheWAS in MGBB further supports domain-specific heterogeneity: women showed associations with obesity, mood and anxiety disorders, and skin cancer, whereas men exhibited ischemic phenotypes (angina pectoris, ischemic heart disease). Such differences might indicate sex-specific circadian genetic risk and hormone-mediated regulation of inflammation.

Importantly, the PheWAS results line up with our genetic correlation and Mendelian randomization results. Significant CII-PRS associations with mood and metabolic traits mirror positive genetic correlation of the CII with MDD, mood swings, BMI, WHR, T2D, CAD, MI, and a strong genetic correlation with insomnia. Mendelian randomization results suggest positive causal associations between the CII and T2D, mood disorders and MI, while reverse MR indicates that genetic liability to CAD, MI and mood disorder can increase CII—consistent with feedback between circadian disruption, inflammation, and cardiometabolic disease. This agreement across methods supports causality over spurious associations due to confounding.

To our knowledge, this study is the first to date to present a genetic analysis of a composite measure of circadian disruption. We proposed the Circadian Imbalance Index (CII), a novel metric that integrates circadian, sleep-related, light-exposure-related, behavioral, and psychological domains. By capturing multiple dimensions of circadian regulation and misalignment, the CII offers a more holistic representation of the underlying biology. This integrative approach has the potential to enhance our understanding of the shared genetic architecture and biological mechanisms governing these interrelated phenotypes and their complex interactions. Our results reflect the pleiotropic nature of circadian imbalance and its potential involvement in diverse biological pathways influencing both mental and physical health and provide genetic evidence reinforcing the relevance of circadian regulation as a potential target for prevention and intervention strategies across multiple domains.

Our study has several limitations that should be acknowledged. First, the analysis was restricted to participants of European ancestry. While this enhances genetic homogeneity and reduces confounding due to population stratification, it also limits the generalizability of our findings to other ancestral groups. Future studies involving more diverse populations are needed to validate and extend our results. Second, except for serum vitamin D levels, the components of the CII were derived from self-reported data, which may be subject to recall bias and misclassification, especially for behavioral measures like caffein consumption or sleep duration. This could potentially attenuate the observed associations. Third, for the purpose of constructing the CII, all component phenotypes were dichotomized, which may have resulted in a loss of information and reduction in statistical power. Fourth, the number of shift workers in our sample was relatively modest and did not allow robust subgroup analyses stratified by both sex and shift-work status, however our findings nevertheless point to loci of interest that warrant further study in larger cohorts. Similarly, our replication cohort was limited to women (N = 11,344), which reduced statistical power to detect interaction effects, and thus we were unable to replicate the shift-work effect modification signals. Nevertheless, of the 104 genome-wide significant lead variants identified in the women-specific UK Biobank GWAS, 84 were represented in the NHS2 replication cohort, and four attained nominal significance, supporting partial consistency and providing a foundation for validation in more highly-powered studies. Furthermore, electronic health records-based codes used in PheWAS aggregate heterogeneous diagnoses and are sensitive to coding bias and biobank-specific recruitment strategies may influence detectability.

In conclusion, our findings demonstrate the value of the CII as a composite genetic metric that captures the multifaceted nature of circadian disruption. We identified novel loci and pathways—including signals modified by sex and shift-work status—underscoring the importance of biological and environmental context in shaping genetic risk. The overlap of CII-associated variants with sleep, psychiatric, metabolic, and cardiovascular traits highlights the broad relevance of circadian imbalance to human health, and our post-GWAS analyses indicate robust associations with T2D, MI and mood disorder. These findings are closely related to the concept of circadian syndrome^73^, which links metabolic syndrome to a broader constellation in which circadian rhythm disorders are a common factor increasing the risk of cardiometabolic and key comorbidities, such as sleep disorders and depression, with links to type 2 diabetes and cardiovascular disease. The observed GSMR effects of CII on T2D and MI, along with links to mood disorders, relate directly to this expanded group of phenotypes, suggesting that genetic susceptibility to circadian rhythm disorders contributes to both metabolic-vascular and psychiatric disorders^73^. The evidence of the reverse direction of CAD/MI/mood on CII is consistent with a bidirectional loop in which cardiometabolic diseases and affective symptoms may further disrupt circadian regulation, reflecting the vicious cycle model embedded within the circadian rhythm syndrome framework.

Clinically, the CII could support risk stratification and earlier referral for sleep and mental-health evaluation and inform screening for metabolic and cardiovascular complications, particularly in shift-working populations. Further studies are needed to validate predictive performance across diverse cohorts and to assess its clinical utility.

## Methods

### UK Biobank

The genetic and phenotypic data for this study were obtained from the UK Biobank^74^ (UKB) under application 48576. The UK Biobank is a large prospective cohort study that began in 2006 and enrolled approximately 500,000 UK residents aged 40 to 69 years. At baseline, participants provided comprehensive data on sociodemographic, psychosocial, physical, and lifestyle factors, along with biological samples. Health outcomes are tracked longitudinally through linkages to national datasets, including Hospital Episode Statistics and national death and cancer registries. The study was approved by the UK National Research Ethics Service (ref. 11/NW/0382), and all participants provided written informed consent.

### Circadian Imbalance Index definition and study population

In our recent study^13^ we developed a novel Circadian Imbalance Index (CII) in the UKB summing up five indicator components increasing individual’s propensity of circadian misalignment (1) evening chronotype, defined as self-reported identification as “definitely an evening person” or “more an evening than a morning person”; (2) short (<7 hours/day) or long (≥9 hours/day) sleep duration; (3) high neuroticism, defined as scoring ≥7 on the neuroticism scale^75,76^; (4) atypical caffeinated coffee consumption, defined as either no intake or high intake (≥5 cups/day); and (5) low serum vitamin D concentration (<50 nmol/L).

Chronotype reflects individual differences in circadian alignment, with morningness (early type) being associated with better alignment^77–79^ and eveningness (late type) being more prone to misalignment^80^. It is strongly **associated with habitual sleep-wake timing**^80,81^, partially predicts meal timing patterns^82,83^ and can therefore serve as a **proxy** for assessing individual differences in sleep- and meal timing. Sleep duration is influenced by the phase of circadian rhythms, with both short (<7 h) and long (>9 h) sleep durations being associated with increased circadian imbalance^84,85^. For neuroticism, strong evidence exist suggesting its link to circadian disruption through shared pathways in stress response, psychiatric disorders, and neurodegeneration^8,86^. Individuals with high neuroticism tend to report more variability in sleep-wake patterns and are more prone to misalignment^87^.

Caffeine directly delays the human circadian clock^21^. In adolescents, its effects on slow-wave sleep (SWS) and melatonin onset vary widely with those showing greater SWS reduction or delayed melatonin being especially vulnerable to sleep–wake desynchronization^88^. As an adenosine receptor antagonist, caffeine’s impact depends on adenosine A2A receptor function and individuals with altered adenosinergic signaling show greater impairment of alertness and readiness during sleep deprivation, linking caffeine sensitivity to circadian resilience^89^. Genetically, caffein sensitivity depends partly on ADORA2A polymorphisms - people with certain variants (TT genotype of rs5751876) experience more anxiety, insomnia, or overstimulation at standard doses and are more likely to avoid coffee^90–92^. Additionally, CYP1A2 variants slow down caffeine metabolism, keeping its levels elevated for longer and increasing symptoms such as palpitations or anxiety, which often leads to self-regulated coffee avoidance^93^. Genetic factors overall explain around 36–58% of individual differences in caffeine consumption, predisposing some to drink less or no coffee^94^. We used “0 cups of caffeinated coffee” as a behavioral proxy for potential caffeine intolerance or genetic hypersensitivity.

Circulating levels of vitamin D serve as a proxy for recent sunlight exposure^95–97^ with low levels being associated with poor sleep quality, short sleep duration^98^ and sleep disorders^99^. This suggests vitamin D’s regulatory role in the sleep–wake cycle^98,100^ further supported by the evidence of its involvement in melatonin production^101,102^, the presence of vitamin D receptors in sleep-related brain regions^98^, as well as by its altering influence on circadian gene expression in adipose-derived stem cells^17^.

Participants not meeting the criteria for a given trait received a score of zero for that component. The CII was calculated as the sum of points across these five domains, yielding a total score ranging from 0 to 5, with higher scores reflecting greater likelihood of circadian imbalance. Further methodological details are provided in Zhang et al^13^.

From the 409,460 UKB participants classified as Europeans using genetically inferred ancestry grouping, as defined by the Pan-UK Biobank^103^, we excluded individuals with missing values (including “prefer not to answer”) at any of the factors used for construction of the CII, leading to a study sample of 312,935 subjects.

### Genome wide association studies in the UKB

#### Overall GWAS

We performed six genome wide association studies (GWASs) - of the five CII components defined as binary phenotypes and the CII itself - with regenie v3.4.1^104^. For quality control, we excluded SNPs with a genotyping efficiency below 80%, minor allele frequencies (MAF) below 1%, or Hardy-Weinberg equilibrium (HWE) values below 1×10^−8^. Further quality control details can be found in Supplementary Methods. We applied linear (for the CII), or logistic (for the CII components) regression models adjusted for age, sex, batch, assessment center and the first 20 genetic principal components. The GWAS model for low vitamin D levels was additionally adjusted for assessment season. We applied a conventional genome wide significance threshold of p<5×10^−8^. For functional annotation we used Functional Mapping and Annotation (FUMA^105^) v1.5.2 (Supplementary Methods). To assess whether the constructed CII captures a meaningful genetic signal, we estimated the SNP-based heritability using Linkage Disequilibrium Score Regression (LDSC)^106^.

#### Genetic correlation analysis

To evaluate the extent to which the CII captures information that is shared with or distinct from its underlying components, we estimated both phenotypic and genetic correlations between the CII and each of its five component traits. Genetic correlations were calculated using LDSC^106^.

#### Genome-wide SNP × Shift Work Interaction Analysis

Shift work status was defined among genotyped European ancestry UKB participants with available CII components (N=312,935) who replied to the baseline questionnaire (2006–2010) and answered the question if their primary job involved shift work (N=183,238), defined as work schedules outside typical daytime hours (9am-5pm). Those who answered with ‘sometimes’, ‘usually’ or ‘always’ (N=28,976) were categorized as ‘shift workers’, whereas those who answered ‘never/rarely’ (N=154,262) were categorized as ‘no shift workers’. Participants who answered with ‘prefer not to answer’ and ‘do not know’ were excluded from this analysis.

To evaluate whether the genetic effects on the CII differ according to the shift work status, we further conducted a genome-wide gene–environment (GxE) interaction analysis. Specifically, we extended the GWAS model to include an interaction term between each SNP and shift work status (SNP × shift work), while adjusting for the same set of covariates as in the primary analysis. For loci reaching genome-wide significance (p < 5 × 10^−8^), we further characterized associations by performing stratified GWAS in shift workers and non-shift workers separately, thereby estimating group-specific effect sizes and directions. In addition, we quantified cross-stratum heterogeneity using a Wald test of the difference in effects and also examined the SNP×SW interaction p-values from the joint model.

#### Replication in the Nurses’ Health Study II

For the aims of replication, we constructed the CII in the women-only, independent study cohort – the Nurses’ Health Study II (NHS2)^26^ – which contained relevant phenotype data. Information on all the five CII components was available for 50,900 European ancestry women. Of them 11,351 had genetic data available. Shift workers in the NHS2 were defined as ever performing shift work (before assessment year 2009). Among 11,351 genotyped women there were 11,344 with shift work assessments; of them N=8,124 (71.6%) nurses were shift workers and N=3,220 (28.4%) never performed shift work before 2009. Socio economic status in the NHS2 was evaluated as in DeVille et.al.^107^ using a composite neighborhood SES (nSES) index derived from nine census tract–level variables capturing education, employment, housing, wealth, racial/ethnic, and population composition, with higher values indicating higher neighborhood SES. Further details on the NHS2 cohort, CII construction, its quality check and description of genetic data in the NHS2 are available in Supplement 6. The NHS2 study protocol was approved by the Institutional Review Boards of the Brigham and Women’s Hospital and the Harvard T.H. Chan School of Public Health.

Since our replication cohort contained only women, we first conducted a genome-wide SNP × sex interaction analysis in the UKB to test whether the genetic effects on the CII differed between men and women. The interaction model included the main effects of the SNP and sex, as well as their product term (SNP × sex) to capture sex-specific modifications of genetic associations. Association testing was carried out genome-wide, and statistical significance of sex-interaction effects was determined based on the p-value of the interaction coefficient. Genome-wide significance was defined at the conventional threshold of p < 5 × 10^−8^. For loci reaching genome-wide significance, we further characterized the associations by conducting follow-up stratified GWASs in men (N=148,473) and women (N= 164,462) separately. We further quantified cross-stratum heterogeneity using a Wald test of the difference in effects and examined the SNP×sex interaction p-values from the joint model^108^.

Replication of CII GWAS in the NHS2 was performed with PLINK 1.9 by assessing effect estimates and p-values from an association test with the CII for variants which attained significance in the UKB-women GWAS. Linear regression models were adjusted for age and 10 first principal components of ancestry.

#### PheWAS in a clinical and population-based biobanks

Further we aimed to determine which external traits and disease phenotypes show association with the CII. We constructed polygenic risk score (PRS) for the CII, overall and stratified by genetic sex, using the PRS-CS^35^ and conducted a phenome-wide association studies (PheWAS) for CII-PRSs in a clinical biobank (the Mass General Brigham Biobank^27^; MGBB) and in a population-based cohort (All Of US^28^; AoU). Associations were considered significant if their p-values were below the Bonferroni corrected significance levels (p_MGBB_<0.05/1,567=3.02E-5; p_AoU_<0.05/933=5.36E-5). Further details on the MGBB, AoU and the PheWAS are described in Supplement 6.

#### Specification of CII associated phenotypes

In order to identify phenotypes associated with CII, we considered (i) GWAS catalog traits and phenotypes linked to gene sets identified in FUMA results and (ii) disease phenotypes identified as significantly associated with the CII-PRS in our PheWAS analyses. For these predefined traits we used UKB-based GWAS summary statistics and calculated their genetic correlation with the CII using LDSC. Those that showed a significant genetic correlation with CII were used in further causality verification.

#### Two sample Mendelian Randomization

To check causality and direction of associations between CII and the predefined list of phenotypes as described above, we performed bidirectional two-sample Mendelian randomization using the generalized summary-data–based Mendelian randomization (GSMR) framework implemented in GCTA^40–42^(v1.94.1). Summary statistics for the CII were obtained from our UK Biobank GWAS, whereas summary statistics for the predefined phenotypes were obtained from non–UK Biobank sources (Table). The MR analysis was run in both directions (Exposure-to-Outcome and Outcome-to-Exposure). We implemented GSMR with HEIDI-outlier filtering (P < 0.01) to mitigate horizontal pleiotropy. Instruments were genome-wide significant variants (P < 5×10^−8^) clumped for independence (LD r^2^ < 0.05) using an LD reference of 425,406 individuals; SNPs with allele mismatches/missingness and large allele-frequency discrepancies versus the LD reference were excluded prior to GSMR. Analyses were required to retain at least 10 instruments after filtering.

## Supplementary Files

This is a list of supplementary files associated with this preprint. Click to download.

- Supplement1TABLESCIIGWAS.pdf
- Supplement2FIGURES.docx
- Supplement3METHODSCIIGWAS.docx
- Supplement4NHS2CIIreplication.docx

## Figures and Tables

**Table 1:** Baseline characteristics of 312,935 genotyped, European ancestry UK Biobank participants with available CII assessments, by CII value and CII components.

		CII value						
		0	1	2	3	4	5	Total
**N (%)**		29,538 (9.4)	87,028 (27.8)	103,761 (33.2)	65,257 (20.9)	23,254 (7.4)	4,097 (1.3)	312,935
**Age** *		58.1 ± 7.8	57.2 ± 8.0	56.5 ± 8.0	55.8 ± 8.1	55.2 ± 8.1	54.2 ± 8.1	56.6 ± 8.1
**Male, N (%)**		15,810 (53.5)	43,253 (49.7)	48,700 (46.9)	29,271 (44.9)	9,781 (42.1)	1,658 (40.5)	148,473 (47.4)
**BMI** *		26.4 ± 3.9	26.9 ± 4.3	27.4 ± 4.7	27.9 ± 5.1	28.5 ± 5.5	29.2 ± 5.9	27.4 ± 4.7
**Average total household income before tax, N (%)**	**Less than £18,000**	4,325 (14.7)	13,504 (15.5)	18,301 (17.7)	13,593 (20.9)	5,979 (25.8)	1,354 (33.3)	5,7056 (18.3)
	**£18,000 to £31,000**	6,602 (22.4)	19,224 (22.1)	22,747 (22.0)	14,478 (22.3)	5,065 (21.9)	861 (21.2)	68,977 (22.1)
	**£31,000 to £52,000**	7,213 (24.4)	21,001 (24.2)	24,690 (23.8)	15,105 (23.2)	5,020 (21.7)	774 (19.0)	73,803 (23.6)
	**£52,000 to £100,000**	6,066 (20.6)	17,882 (20.6)	20,307 (19.6)	11,522 (17.7)	3,446 (14.9)	487 (12.0)	59,710 (19.1)
	**Greater than £100,000**	1,952 (6.6)	5,221 (6.0)	5,458 (5.3)	2631 (4.0)	750(3.2)	87(2.1)	16,099 (5.2)
	**Unknown**	3,352 (11.4)	10,070 (11.6)	12,040 (11.6)	7,730 (11.9)	2,879 (12.4)	504 (12.4)	36,575 (11.7)
**Education, N (%)**	**College or University degree**	10,544 (35.7)	30,398 (34.9)	34,399 (33.2)	19,617 (30.1)	6,266 (26.9)	980 (23.9)	102,204 (32.7)
	**Other**	14,788 (50.1)	43,294 (49.7)	52,267 (50.4)	33,678 (51.6)	12,073 (51.9)	2,168 (52.9)	158,268 (50.6)
	**Unknown**	4,206 (14.2)	13,336 (15.3)	17,095 (16.5)	11,962 (18.3)	4,915 (21.1)	949 (23.2)	52,463 (16.8)
**Shift workers, N (%** ^(a)^ **)**		2,020 (13.0)	6,938 (13.8)	9,628 (15.4)	7,009 (17.8)	2,821 (21.0)	560 (26.3)	28,976 (9.3)
		CII components						
		Evening Person	Short Or Long Sleep	High Neuroticism	No Or High Caffeine	Low Vit D	Total	
**N (%)**		105,673 (33.8)	96,903 (31.0)	74,742 (23.9)	161,085 (51.5)	165,419 (52.9)	312,935	
**Age** *		55.7 ± 8.3	57.1 ± 7.9	55.3 ± 8.0	56.2 ± 8.1	56.0 ± 8.1	56.6 ± 8.1	
**Male, N (%)**		49,943 (47.3)	46,105 (47.6)	28,978 (38.8)	72,382 (44.9)	78,472 (47.4)	148,473 (47.4)	
**BMI** *		27.6 ± 4.8	28.0 ±5.1	27.4 ±5.1	27.5 ± 4.9	28.0 ± 5.1	27.4 (4.7)	
**Average total household income before tax, N (%)**	**Less than £18,000**	19,374 (18.4)	22,205 (23.0)	16,942 (22.7)	31,267 (19.5)	31,783 (19.3)	57,056 (18.3)	
	**£18,000 to £31,000**	23,142 (22.0)	21,574 (22.3)	16,478 (22.1)	35,864 (22.3)	35,659 (21.6)	68,977 (22.1)	
	**£31,000 to £52,000**	25,258 (24.0)	20,912 (21.7)	16,888 (22.7)	37,342 (23.2)	39,246 (23.8)	73,803 (23.6)	
	**£52,000 to £100,000**	20,642 (19.6)	15,169 (15.7)	12,375 (16.6)	29,217 (18.2)	31,878 (19.3)	59,710 (19.1)	
		CII value						
		0	1	2	3	4	5	Total
	**Greater than £100,000**	5,507 (5.2)	3,945 (4.1)	2,631 (3.5)	7,147 (4.4)	8,235 (5.0)	16,099 (5.2)	
	**Unknown**	11,505 (10.9)	12,746 (13.2)	9,170 (12.3)	19,818 (12.3)	18,137 (11.0)	36,575 (11.7)	
**Education, N (%)**	**College or University degree**	35,511 (33.6)	26,268 (27.1)	21,327 (28.5)	47,558 (29.5)	57,347 (34.7)	102,204 (32.7)	
	**Other**	54,562 (51.6)	49,737 (51.3)	38,740 (51.8)	83,752 (52.0)	81,203 (49.1)	158,268 (50.6)	
	**Unknown**	15,600 (14.8)	20,898 (21.6)	14,675 (19.6)	29,775 (18.5)	26,869 (16.2)	52,463 (16.8)	
**Shift workers, N (%**^(a)^)		11,082 (17.4)	10,515 (19.9)	7,504 (17.3)	15,835 (16.6)	16,369 (16.0)	28,976 (9.3)	

N: sample size; BMI: Body mass index;

* Values are **means ± standard deviations;**

(a) Percentages among N = 183,238 genotyped European ancestry UKB participants with all CII components available, who replied to the baseline questionnaire (2006–2010) and answered to the question if their primary job involved shift work (defined as work schedules outside 9am-5pm). Shift workers are those who answered with ‘sometimes’, ‘usually’ or ‘always’. Participants who answered with ‘prefer not to answer’ and ‘do not know’ were excluded from this analysis

**Table 2:** Genomic risk loci resulting from FUMA analysis based on the GWAS of the CII among 312,935 European ancestry UK Biobank participants.

Genomic Locus	Variant	Chromosome: position	Nearest gene	Effect allele	Effect size (P)	Standard error	P-value
7	rs34265662	4:72617557	GC	TA	0,0793	0,0031	1,49E-147
11	rs117913124	11:14900931	CYP2R1	A	0,2192	0,0085	1,01E-146
14	rs3794060	11:71187679	NADSYN1	T	−0,0434	0,0034	5,05E-37
10	9:140259068_T_TA	9:140259068	EXD3	TA	0,0301	0,0043	1,57E-12
9	rs73198970	8:11040216	XKR6	C	0,0187	0,0028	3,47E-11
12	11:57522200_CCCCT_C	11:57522200	TMX2-CTNND1:RP11-691N7.6:CTNND1	C	0,0196	0,0030	6,42E-11
22	rs567979837	13:60753105	RNY4P28	CT	−0,0185	0,0028	7,51E-11
25	rs1557341	18:35127427	CELF4	C	−0,0193	0,0030	9,28E-11
5	rs7652808	3:85603643	CADM2	G	0,0185	0,0029	2,12E-10
21	rs10859995	12:96375682	HAL	C	0,0178	0,0028	3,08E-10
27	rs2547244	19:48363039	TPRX2P	G	0,0231	0,0037	4,92E-10
17	rs6487365	12:24066460	SOX5	A	0,0183	0,0030	9,12E-10
26	rs2126786	18:53447005	RP11-397A16.1:RP11-397A16.2	C	0,0225	0,0037	1,01E-09
19	12:39098718_GT_G	12:39098718	CPNE8	G	0,0171	0,0028	1,55E-09
16	rs10848644	12:365289	SLC6A13:RP11-283I3.4	T	−0,0167	0,0028	2,81E-09
1	rs61816761	1:152285861	FLG-AS1:FLG	A	−0,0574	0,0097	3,80E-09
8	rs2979241	8:8303353	CTA-398F10.2	C	−0,0162	0,0028	7,34E-09
3	rs11563179	2:51693002	AC007682.1	C	0,0222	0,0039	9,43E-09
2	rs2279681	1:201861016	SHISA4	G	−0,0168	0,0029	1,02E-08
20	rs139923919	12:93222395	EEA1	CAT	−0,0176	0,0031	1,04E-08
13	rs509533	11:66070575	TMEM151A	C	0,0159	0,0028	1,68E-08
23	rs16661	14:75128316	AREL1	C	0,0165	0,0030	2,74E-08
18	rs9668760	12:34611172	RP13-7D7.1	G	−0,0157	0,0028	2,98E-08
24	rs62007299	15:77711719	PEAK1	A	0,0169	0,0031	3,55E-08
4	rs7625384	3:18774357:G:T	AC144521.1	T	0,0170	0,0031	3,89E-08
6	rs4082244	3:134729537:C:G	EPHB1	G	−0,0160	0,0029	4,79E-08
15	rs10750539	11:133548873:A:G	RP11-448P19.1	A	0,0160	0,0029	4,81E-08

**Table 3:** Circadian rhythm related genes resulting from FUMA analysis based on the GWAS of the CII among 312,935 European ancestry UK Biobank participants.

Ensg	Symbol	Chr	Strand	MsigDB Label	PLI	Min GWAS p	Ind Sig Snps	GL
ENSG00000110680	CALCA	11	−1	WP CLOCKCONTROLLED AUTOPHAGY IN BONE METABOLISM^(a)^	0.0014	4.8730e-25	11:14992795_AGGAGCCCACAGACCTT_A; rs61878675	11
ENSG00000172893	DHCR7	11	−1	UEDA PERIFERAL CLOCK^(b)^	5.0427e-08	6.1235e-37	rs12793607, rs1540129, rs3794060, rs1790349, rs369124946, 11:71203933_AT_A, rs181766110, rs139168803, rs35708376, rs760165576	14
ENSG00000073614	KDM5A	12	−1	WP CIRCADIAN RHYTHM GENES^(c)^	0.99999	6.1331e-07	rs10848644	16
ENSG00000084110	HAL	12	−1	UEDA PERIFERAL CLOCK^(b)^	5.9087e-17	3.0841e-10	rs10859995	21
ENSG00000105392	CRX	19	1	WP CIRCADIAN RHYTHM GENES^(c)^	0.6237	4.7685e-08	rs2547244	27

**Ensg**: Ensembl gene identifier; **Chr**: chromosome; **MsigDB Label**: Molecular Signatures Database label; **PLI**: probability of being loss-of-function intolerant; **Min GWAS p**: minimum p-value from GWAS analysis; I**nd Sig Snps**: Independent significant SNPs; **GL**: Genomic Loci.

(a) systematic name: M45523; MSigDB url: https://www.gsea-msigdb.org/gsea/msigdb/human/geneset/WP_CLOCKCONTROLLED_AUTOPHAGY_IN_BONE_METABOLISM;

(b) systematic name: M12498; MSigDB url: https://www.gsea-msigdb.org/gsea/msigdb/human/geneset/UEDA_PERIFERAL_CLOCK; PMID: 15273285

(c) systematic name: M39605; MSigDB url: https://www.gsea-msigdb.org/gsea/msigdb/human/geneset/WP_CIRCADIAN_RHYTHM_GENES.

**Table 4:** Genes significantly associated with the CII but not with any of its individual components, obtained from FUMA analysis based on the GWAS of the CII among 312,935 European ancestry UK Biobank participants together with effect sizes (β), standard errors (se) and p-values (Ind Sig SNP p) for the corresponding independent significant SNPS in an overall analysis (GWAS overall) and among N = 164,462 European ancestry UK Biobank women (GWAS in women).

						GWAS overall			GWAS in women		
Ensg	GL	Symbol	Chr	PLI	Ind Sig Snps	Ind Sig SNP p	β	se	Ind Sig SNP p	β	se
ENSG00000154928	6	EPHB1^(a), (b)^	3	9.98E-01	rs4082244	4.79E-08	−0.0160	0.0029	1.47E-07	−0.0303	0.0058
ENSG00000149131	12	SERPING1^(a), (b), (d)^	11	9.73E-01	rs1647396	2.46E-07	0.0153	0.0028	-	-	-
ENSG00000214215	20	C12orf74^(a), (b)^	12	5.22E-04	rs139923919	1.04E-08	−0.0176	0.0031	5.13E-06	0.0178	0.0039
ENSG00000187510	20	PLEKHG7^(a), (b)^	12	3.68E-11	rs139923919						
ENSG00000102189	20	EEA1^(a), (c)^	12	6.97E-01	rs139923919						

**Ensg**: Ensembl gene identifier; **Chr**: chromosome; **MsigDB Label**: Molecular Signatures Database label; **GL**: Genomic Loci; **PLI**: probability of being loss-of-function intolerant; I**nd Sig Snps**: Independent significant SNPs; **Ind Sig SNP p**: p-value for Ind Sig SNP;

(a) Protein coding;

(b) forward strand;

(c) reverse strand;

(d) gene did not appear in women-specific FUMA results.

## Data Availability

The data underlying this article cannot be shared publicly. However, researchers are encouraged to apply to access to the UK Biobank resource for health-related research that serves the public interest.
